# Assessing the Availability of Data on Social and Behavioral Determinants in Structured and Unstructured Electronic Health Records: A Retrospective Analysis of a Multilevel Health Care System

**DOI:** 10.2196/13802

**Published:** 2019-08-02

**Authors:** Elham Hatef, Masoud Rouhizadeh, Iddrisu Tia, Elyse Lasser, Felicia Hill-Briggs, Jill Marsteller, Hadi Kharrazi

**Affiliations:** 1 Center for Population Health IT Department of Health Policy and Management Johns Hopkins Bloomberg School of Public Health Baltimore, MD United States; 2 Johns Hopkins Center for Health Disparities Solutions Baltimore, MD United States; 3 Center for Clinical Data Analysis Institute for Clinical and Translational Research Johns Hopkins School of Medicine Baltimore, MD United States; 4 Division of Health Sciences Informatics Johns Hopkins School of Medicine Baltimore, MD United States; 5 Department of Medicine Johns Hopkins School of Medicine Baltimore, MD United States; 6 Department of Health, Behavior, and Society Johns Hopkins Bloomberg School of Public Health Baltimore, MD United States; 7 Department of Acute and Chronic Care Johns Hopkins School of Nursing Baltimore, MD United States; 8 Welch Center for Prevention, Epidemiology & Clinical Research Johns Hopkins University Baltimore, MD United States; 9 Behavioral, Social and Systems Sciences Translational Research Community Institute for Clinical and Translational Research Johns Hopkins School of Medicine Baltimore, MD United States; 10 Center for Health Services and Outcomes Research Department of Health Policy and Management Johns Hopkins Bloomberg School of Public Health Baltimore, MD United States; 11 Armstrong Institute for Patient Safety and Quality Johns Hopkins School of Medicine Baltimore, MD United States

**Keywords:** social and behavioral determinants of health, electronic health record, structured data, unstructured data, natural language processing, multi-level health care system

## Abstract

**Background:**

Most US health care providers have adopted electronic health records (EHRs) that facilitate the uniform collection of clinical information. However, standardized data formats to capture social and behavioral determinants of health (SBDH) in structured EHR fields are still evolving and not adopted widely. Consequently, at the point of care, SBDH data are often documented within unstructured EHR fields that require time-consuming and subjective methods to retrieve. Meanwhile, collecting SBDH data using traditional surveys on a large sample of patients is infeasible for health care providers attempting to rapidly incorporate SBDH data in their population health management efforts. A potential approach to facilitate targeted SBDH data collection is applying information extraction methods to EHR data to prescreen the population for identification of immediate social needs.

**Objective:**

Our aim was to examine the availability and characteristics of SBDH data captured in the EHR of a multilevel academic health care system that provides both inpatient and outpatient care to patients with varying SBDH across Maryland.

**Methods:**

We measured the availability of selected patient-level SBDH in both structured and unstructured EHR data. We assessed various SBDH including demographics, preferred language, alcohol use, smoking status, social connection and/or isolation, housing issues, financial resource strains, and availability of a home address. EHR’s structured data were represented by information collected between January 2003 and June 2018 from 5,401,324 patients. EHR’s unstructured data represented information captured for 1,188,202 patients between July 2016 and May 2018 (a shorter time frame because of limited availability of consistent unstructured data). We used text-mining techniques to extract a subset of SBDH factors from EHR’s unstructured data.

**Results:**

We identified a valid address or zip code for 5.2 million (95.00%) of approximately 5.4 million patients. Ethnicity was captured for 2.7 million (50.00%), whereas race was documented for 4.9 million (90.00%) and a preferred language for 2.7 million (49.00%) patients. Information regarding alcohol use and smoking status was coded for 490,348 (9.08%) and 1,728,749 (32.01%) patients, respectively. Using the International Classification of Diseases–10th Revision diagnoses codes, we identified 35,171 (0.65%) patients with information related to social connection/isolation, 10,433 (0.19%) patients with housing issues, and 3543 (0.07%) patients with income/financial resource strain. Of approximately 1.2 million unique patients with unstructured data, 30,893 (2.60%) had at least one clinical note containing phrases referring to social connection/isolation, 35,646 (3.00%) included housing issues, and 11,882 (1.00%) had mentions of financial resource strain.

**Conclusions:**

Apart from demographics, SBDH data are not regularly collected for patients. Health care providers should assess the availability and characteristics of SBDH data in EHRs. Evaluating the quality of SBDH data can potentially enable health care providers to modify underlying workflows to improve the documentation, collection, and extraction of SBDH data from EHRs.

## Introduction

### The Role of Social and Behavioral Determinants of Health in Changing US Health Care System

The US health care system is moving toward *pay for performance* and value-based incentive programs [1]. To be eligible for value-based programs and to improve the quality of care while reducing cost, health care providers need to assess social and behavioral determinants of health (SBDH) for both patients and populations [1]. SBDH are “the conditions in which people are born, grow, work, live, and age, also the wider set of forces and systems shaping the conditions of daily life” [2]. SBDH are powerful drivers of morbidity, mortality, and future well-being of individuals and communities [3]. Without considering SBDH factors in decision making and program development, the special needs of high-cost patients who are concomitantly facing socioeconomic challenges and behavioral health problems might not be properly addressed, thus resulting in poor outcomes and financial penalties for providers [4].

### Challenges Related to Accessing Data on Social and Behavioral Determinants of Health

Despite the importance and significant impact of SBDH on utilization and outcomes, medical care providers often rely on administrative claims to assess SBDH data, which tend to lack information on important determinants affecting health [3]. Health care systems seeking access to SBDH data through their electronic health records (EHRs) face various challenges in searching and summarizing structured and unstructured data (clinical free-text notes) [5-7]. Although some EHR vendors have started adding specific fields for collecting SBDH data, no universally accepted and standardized format exists for documenting SBDH data in EHRs’ structured data. In addition, extracting data from unstructured EHR data requires time-consuming and subjective methods, such as chart review, which is not a feasible approach to screen a large population of patients [5-9].

In 2014, to address the lack of SBDH data collection by health care providers, the National Academy of Medicine (NAM) recommended a set of social and behavioral domains and measures for EHRs [10,11]. Meanwhile, clinical informaticians and health information technology experts have started to assess and optimize the documentation and collection of SBDH data in EHRs for specific subpopulations of patients [12-17]. Although these initial efforts are promising, previous studies lack an in-depth assessment of SBDH data documentation, collection, and presentation within a major health system’s EHR using both structured and unstructured fields.

Several states, including Maryland, have begun to incentivize health care systems to find cost-effective solutions that improve population health in their communities [18,19]. In this context, leveraging data on SBDH is essential for providers to improve the quality of care, reduce health care costs, and meet the requirements of these newly developed SBDH-adjusted reimbursement models [20]. To address this need, we aimed to examine the availability and characteristics of SBDH data in EHR’s structured data of a multilevel academic health care system with linked ambulatory provider networks in Maryland. We also assessed the feasibility of using text mining—a natural language processing (NLP) technique—to extract SBDH data from EHR’s unstructured data [12,13,21].

## Methods

### Data Source

We extracted EHR data from a multilevel academic health care system with linked ambulatory provider networks providing services to patients with varying SBDH (eg, different levels of socioeconomic status) across Maryland. The EHR contained data migrated from previous EHR systems in different facilities across the health care system from 2003 to 2018 (see Multimedia Appendix 1). EHR migration started in 2013 and finished by 2016, with all facilities having full access to the same EHR platform. We used the EHR as the sole data source for this study and excluded any legacy or ancillary systems (eg, administrative systems) because of variations of such ancillary systems across health systems.

The structured data included in this study represented information collected between January 2003 and June 2018 from 5,401,324 unique patients. We also used the EHR’s unstructured data of 1,188,202 unique patients captured between July 2016 (when all facilities had full access to the EHR and thus the potential to record unstructured data) and May 2018 (when this study was completed).

### Selected Social and Behavioral Domains

SBDH can be defined as characteristics of patients and communities. The NAM recommends that certain patient-level SBDH domains be collected in EHRs for use in clinical practice (see Multimedia Appendix 2) [10,11]. We narrowed the NAM list of patient-level SBDH domains after conducting a comprehensive literature review, consulting with clinicians and researchers who collect and use the SBDH data regularly, gauging the basic availability of domain-specific SBDH factors in the EHR, and high-level priorities of the health care system [22]. SBDH domains assessed in this study included the following: (1) patient address/zip code, (2) ethnicity, (3) race, (4) preferred language, (5) alcohol use presented as the number of alcoholic drinks per week, (6) smoking status, (7) social connection/isolation, (8) housing issues, and (9) income/financial resource strain. Except for patients’ address and location that could be tied into community-level SBDH, all SBDH factors assessed in this study were considered patient-level.

Using the definition provided by the NAM [11], we defined social connection as the degree to which a person has social ties or relationships with other individuals, groups, or organizations. Social isolation would be a state of loneliness with lack of interaction with others and those detached and isolated with no help or support system. For assessment of housing issues, we categorized them into those related to homelessness, inadequate housing (housing instability or insecurity), and housing characteristics (quality and characteristics of the building of patient’s residence). We defined patients with income/financial resource strain as those in deteriorated financial status, financial hardship, or in poverty (eg, unable to afford the basics of life and/or medical interventions and in need and eligible for any benefit or enrollment in financial assistance programs). Financial resource strain reflected the absence of sufficient resources as well as the lack of an individual’s skills and knowledge needed to manage resources.

### Structured Data Analysis

In a previous study, our study team developed a series of data collection metrics to capture information of interest [22], which included the following: (1) most common collection method (eg, standardized EHR-provided data elements, such as diagnosis and procedures as well as custom-made EHR-embedded structured questionnaires), (2) completeness rate, (3) collection date range, (4) facility type and collection location (eg, inpatient and outpatient), and, (5) type of providers who recorded the data (eg, physician, nurse, social worker, and case manager). For data elements captured in EHR-provided data fields or EHR-embedded questionnaires, we used structured query language (SQL)—a standard language for storing, manipulating, and retrieving data in databases—to find instances of data domains (eg, *housing* or *social support*). We also used SQL to tabulate patient counts, encounters, locations, and providers. For data variables associated with International Classification of Diseases–10th Revision (ICD-10)–coded diagnoses, we used a built-in EHR tool [23] to return counts of unique patients.

### Unstructured Data Analysis

We explored the use of text-mining techniques, such as pattern matching, to determine SBDH from the EHR’s unstructured data [14]. To identify notes containing those determinants, we used handcrafted linguistic patterns that a team of experts developed using ICD-10, current procedure terminology, logical observation identifiers names and codes (LOINC), and systematized nomenclature of medicine (SNOMED) terminologies [24,25] and the description of those determinants in public health surveys and instruments (eg, American Community Survey [26], American Housing Survey [27], The Protocol for Responding to and Assessing Patients’ Assets, Risks, and Experiences [28], and the Accountable Health Communities tool from the Center for Medicare and Medicaid Innovation [29]). We also reviewed phrases derived from a literature review of other studies and the results of a manual annotation process from a previous study [12,30].

To craft the linguistic patterns, the expert team focused on 3 domains (social connection/isolation, housing issues, and income/financial resource strain) and developed a comprehensive list of all available codes and specific content areas for each selected domain and matched them across different coding systems. Multimedia Appendices 3 and 4 present examples of available codes for different subdomains of housing issues and example of phrases developed for social connection/isolation.

To assess the accuracy of the information retrieved through text-mining techniques, we performed a manual annotation of 100 randomly selected notes for subdomain of homelessness within the housing SBDH domain.

The Institutional Review Board of Johns Hopkins Bloomberg School of Public Health approved this study.

## Results

### Social and Behavioral Domains Extracted From Structured Data

Table 1 presents collection methods and characteristics of selected domains in the EHR’s structured data. Of approximately 5.4 million unique patients, we identified demographic data for a large number but only 490,348 patients (9.08%) reported information regarding alcohol use with 178,789 (3.31%) patients reporting one or more drinks per week. In addition, 1,728,749 patients (32.01%) reported smoking status in their social history.

**Table 1:** Collection methods and characteristics of selected social and behavioral determinants of health in electronic health records’ structured data^a^.

Common collection method		Completeness rate	Collection date	Facility type	History and details	Other collection methods^b^
**Patient address/zip code**						
	Upon registration of each encounter. Documented as a street name and number, an optional line for apartment or other information, a city, a state or province, and a zip code.	Approximately 5.2 million patients (95%)	2003-Current	All facilities at the time of registration	Approximately 66% of patients’ address change records are available, with effective start and end dates to track address change over time	Billing address, claims processing address, home health encounters and episodes, communications for specific encounters
**Ethnicity**						
	Upon registration of each encounter	Approximately 2.7 million patients (50%)	2003-Current	All facilities at the time of registration	Ethnicity (Hispanic or non-Hispanic) captured separately from race	Transplant organ donors, ethnicity questionnaire, ethnicity origin questionnaire
**Race**						
	Upon registration of each encounter	Approximately 4.9 million patients (90%) indicated at least one race	2003-Current	All facilities at the time of registration	Patients can self-identify multiple races	Home health, transplant organ donors
**Preferred language**						
	At the time of admission	2,718,416 patients (50%)	2003-Current	All facilities at the time of an encounter	The top preferred languages, by unique patient count: English (2,626,379, 48.6%) and Spanish (53,446, 0.9%)^c^	Flowsheets, questionnaires, clinical notes
**Alcohol use: alcoholic drinks per week**						
	Social history portion of electronic health record during a patient encounter, whether in-person or not in-person encounters (telephone, MyChart^d^, documentation)	490,348 (9.08%) patients, 178,789 (3.31%) patients reported one or more drinks per week	2013-Current	All facilities at the time of an encounter	Reports show having any value (including 0 alcoholic drinks per week) in social history	Flowsheets, questionnaires, clinical notes
**Smoking status**						
	Social history portion of electronic health record during a patient encounter, whether in-person or not in-person encounters (telephone, MyChart^d^, documentation)	1,728,749 (32%) patients reported having any value smoking status in social history	2013-Current	All facilities at the time of an encounter	Smoking quit date is also populated but only in 137,958 (2.6%) of encounters^e^	Flowsheets, questionnaires, clinical notes

^a^Structured electronic health record data were collected from approximately 5.4 million unique patients between January 1, 2003 and June 26, 2018 and data on alcohol use and smoking status were collected since April 2013.

^b^The highest completion rate among other collection methods. The complete list and characteristics of other collection methods are available in Multimedia Appendix 5.

^c^Other preferred languages were—Arabic: 7317 (0.14%), Chinese/Mandarin: 4036 (0.07%), Korean: 3168 (0.06%), Unknown—a valid value in EHR, different from an empty record: 5936 (0.11%), and no language reported: 2,804,973 (51.93%).

^d^Integrated patient portal of the electronic health record system.

^e^The status breakdown with collection rate was—current every day smoker: 114,566 (2.12%), current some day smoker: 28,547 (0.53%), former smoker: 297,099 (5.5%), heavy tobacco smoker: 3111 (0.06%), light tobacco smoker: 12,857 (0.24%), never assessed: 302,631 (5.60%), never smoker: 952,636 (17.64%), passive smoke exposure/never smoker: 4274 (0.08%), ever smoked/current status unknown: 1133 (0.02%), and unknown if ever smoked: 11,915 (0.22%).

Table 2 presents counts and percentages of patients having ICD-10– or equivalent ICD-9–coded diagnoses for selected domains on their problem lists, in their EHR-derived billing codes, or recorded at the time of an encounter. The diagnoses-based query results used the same denominator as Table 1 (approximately 5.4 million unique patients), among whom there were a few patients with information related to social connection/isolation (35,171; 0.65%), housing issues (10,433; 0.19%), and income/financial resource strain (3543; 0.07%). Counts and percentages of patients having any of these SBDH within the unstructured data were calculated based on approximately 1.2 million unique patients denominator. The NLP technique did not distinguish the subtypes of each SBDH, hence counts and percentages for specific ICD Z codes are missing for unstructured data.

Several questionnaires were identified in the EHR data warehouse that captured information on selected SBDH domains. Table 3 presents a select list of questionnaire templates, content areas, total number of completed questionnaires, and the percentage of answered questions related to the selected domains. The characteristics of questionnaires are provided in Multimedia Appendix 6. The list of questionnaires is not exhaustive but represents most questionnaires in the EHR under study that were available as of July 2018. Note that a patient may fill a questionnaire more than once, hence the number of administered or completed questionnaires does not necessarily translate into the number of patients having a certain SBDH. We could not calculate the number of unique patients represented by the questionnaires because of various study protocols using internal identity documents linking questionnaire results to patients, which were inaccessible in our study.

**Table 2:** Number of patients with selected social and behavioral determinant of health (SBDH) domains in electronic health records—using diagnoses-based query and unstructured data.

SBDH categories and subtypes/codes^a^		Diagnoses-based query, patient count^b^	Unstructured, patient count^c^
**Social connection/isolation, n (%)**		31,628 (0.58)	30,893 (2.59)^d^
	Z60.2 problems related to living alone, n	1222	—^e^
	Z60.4 social exclusion and rejection, n	223	—
	Z63.0 relationship problems (with spouse/partner), n	852	—
	Z63.5 family disruption (separation/divorce), n	548	—
	Z63.8 other primary support group problems, n	2230	—
	Z63.9 unspecified primary support group problem, n	3247	—
	Z65.9 unspecified psychosocial circumstances, n	938	—
	Z73.4 inadequate social skills, n	81	—
	Z91.89 other specified personal risk factors, n	18,947	—
	R45.8 other emotional state symptoms and signs, n	3340	—
**Housing issues, n (%)**		10,433 (0.19)	35,646 (2.99)^d^
	Z59.0 homelessness, n	7022	—
	Z59.1 inadequate housing, n	120	—
	Z59.8 other housing problems, n	3291	—
**Income/financial resource strain, n (%)**		3543 (0.06)	11,882 (0.99)^d^
	Z59.5 extreme poverty, n	68	—
	Z59.6 low income, n	72	—
	Z59.7 insufficient social insurance and welfare, n	46	—
	Z59.8 other economic circumstances problems, n	3357	—

^a^Patients with international classification of diseases–revision 9 and 10–coded diagnoses were included in the query.

^b^Structured electronic health record data were collected from approximately 5.4 million unique patients that contained information captured from January 1, 2003 through June 26, 2018.

^c^Unstructured data were captured between July 1, 2016 and May 31, 2018. The notes represented 1,188,202 unique patients and 9,066,508 unique encounters.

^d^Number of unique patients with at least one note with mentions of the selected social and behavioral domain. Subcategories of social connection/isolation and income/financial resource strains were not studied separately using unstructured data.

^e^Data not available.

**Table 3:** Characteristics of electronic health record questionnaires for selected social and behavioral determinant of health domains.

Questionnaire template			Content area	Administered questionnaires^a^, completed, n (%)
**Social support**				
	Nursing assessment (n^b^=1,026,988)		Psychological-social relationship	944,829 (92.00)
	**Emergency department assessment**			
		Head-to-toe (n=237,143)	Psychological-social relationship	92,486 (39.00)
		Nursing 1 (n=217,954)	Psychological-social relationship	204,877 (94.00)
		Nursing 2 (n=278,084)	Psychological-social relationship	169,631 (61.00)
		Pediatrics (n=131,134)	Psychological-social relationship	93,105 (71.00)
		Social work suicide/homicide (n=15,101)	Relationship and social support status	14,648 (97.00)
		Social work (n=14,481)	Support system’s name and information	12,743 (88.00)
	Operation room and post anesthesia care unit flowsheet (n=147,694)		Psychological-social relationship	82,709 (56.00)
	**Inpatient**			
		Occupational therapy new home setup (n=131,948)	Social support available at discharge	47,501 (36.00)
		Obstetrics postpartum assessment (n=135,587)	Recent loss or change in status	120,672 (89.00)
		Spiritual care interventions (n=116,719)	Spiritual/social network	68,864 (59.00)
	Pediatrics screening (n=144,659)		Personal-social relationship or socially withdrawn and decreased interaction	85,349 (59.00)
	Social history; screening, brief intervention, and referral to treatment (n=2015)		Marital status/need to improve relationships with family/social network and participation in social activities	1995 (99.00)
**Housing issues**				
	Housing/utility voucher (n=217)		Housing assistance screening and referral	97 (44.00)
	Abuse/neglect screen (n=12,058)		Homelessness assessment	11,575 (96.00)
	Social history questionnaire (n=1900)		Screening for assistance with finding housing	1824 (96.00)
	Emergency department triage abuse indicators and resource planning (n=713,702)		Information on shelter, transportation, and clothing	39,254 (5.50)
	Chemical dependence unit admission screen (n=15,056)		Homelessness	2258 (15.00)
	Ambulatory priority access primary care screen (n=1116)		Housing situation	78 (7.00)
	Adult admission general intake form (n=77,230)		Homelessness	27030 (35.00)
	Pediatric/newborn general intake form (n=1067)		Homelessness	587 (55.00)
	Psychiatry social work assessment (n=4913)		Living arrangement	4422 (90.00)

^a^Represents completed questionnaires (count and % of answered questions related to social and behavioral domain of interest). The timeframe for questionnaires was January 1, 2003 to June 26, 2018, with approximately 5.4 million unique patients.

^b^Represents total number of questionnaires available on electronic health record.

### Selected Social and Behavioral Domains Extracted From Unstructured Data

**Figure 1:**
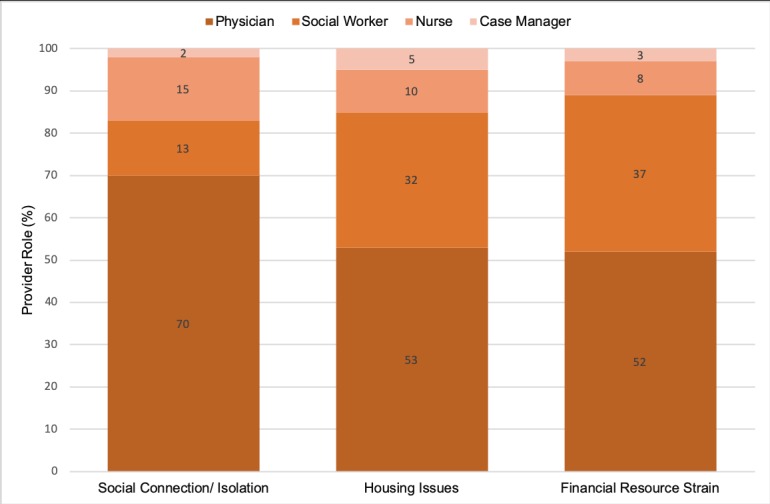
Characteristics of the electronic health record's unstructured data containing social and behavioral determinants of health, stratified by provider role.

**Figure 2:**
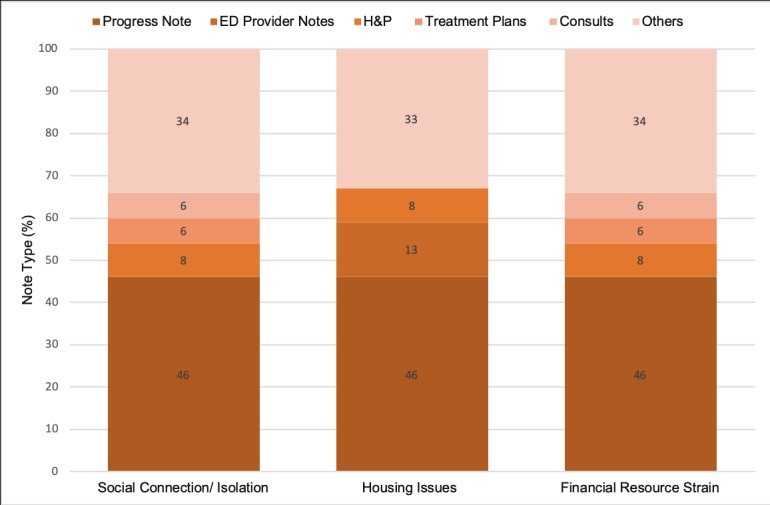
Characteristics of the electronic health record's unstructured data containing social and behavioral determinants of health, stratified by note type.

We used NLP (ie, text-mining techniques) to identify select SBDH domains available from the EHR’s unstructured data represented by 9,066,508 unique encounters spanning from July 1, 2016 to May 31, 2018. Of 1,188,202 unique patients, 2.6% had at least one note containing social connection/isolation, 3.0% had mention of housing issues, and 1.0% had at least one note with a phrase about income/financial resource strain (see Table 2). Notes containing mentions of SBDH were generated by several provider roles across different facilities and collected for various encounter types (see Figures 1 and 2). Physicians recorded most of the information for the selected SBDH domains. Progress notes contained most of the phrases reflecting the selected SBDH domains.

The manual annotation of 100 randomly selected notes for subdomain of homelessness within the housing SBDH domain showed that the word *homeless* appeared 130 times: 64 notes contained true positive mentions, 14 notes contained false positive mentions, 20 notes contained true negative mentions, and 2 notes contained conflicting true positive and false positive mentions of the phrase *homeless* within the same note. The 20 notes containing true negative mentions were derived from EHR’s *SmartPhrases*, which are automatically generated phrases after a few characters are typed, available in specific contexts, such as questionnaires. In our sample notes, the SmartPhrases contained the question *Is Patient Homeless?* with the *Yes or No* answer for providers to choose. The provider’s answer to the SmartPhrases question was no for all 20 cases. We did not identify any false negative phrases. Identification of those phrases requires manual annotation of SBDH in a large body of text, which will be conducted in the next phase of this study.

## Discussion

### Overall Findings

Despite the significant impact of SBDH on health outcomes, health care providers rarely have standardized tools available to systematically collect and incorporate information about SBDH factors into decision making, program development, and adjustment of payment models [3]. Most SBDH data are not discretely represented or captured in structured formats in EHRs. Despite ongoing efforts to use NLP techniques for data extraction on SBDH from unstructured free text (eg, clinical notes), off-the-shelf data extraction solutions are lacking for SBDH data in contrast to clinical diagnostic codes and their standardized terminology [5,7]. Standardized EHR-based tools for collection of SBDH data could lead to improved patient and population health outcomes in different care settings [31]. An assessment of availability and characteristics of SBDH data in EHRs of health care systems, such as the one presented in this study, can be the first step for developing such SBDH data extraction tools.

In this study, we analyzed the capture rate of SBDH data within our EHR system for a range of SBDH domains. To achieve this goal, we assessed various sources of data within the EHR: structured fields, embedded questionnaires, and unstructured free text, such as clinical notes (see Multimedia Appendix 5 for additional details). Our findings showed high to moderate rates of data collection, ranging from 49% to 95%, for select SBDH domains (eg, valid address/zip, race, ethnicity, and preferred language) using EHR’s structured data. However, we identified modest to low rates of documented information on other SBDH domains, such as drinking habits and smoking status (ranging from 9% to 32%). We also explored more complex SBDH domains using coded diagnoses and found very low rates of data captured for social connection/isolation, housing issues, or income/financial resource strain (all factors <0.7%). Applying NLP techniques, such as text mining, on EHR’s unstructured data, however, identified additional patients with social connection/isolation, housing issues, or income/financial resource strain (rates ranging from 1% to 3%).

### Comparing With Previous Studies

Previous studies using EHR’s structured fields to extract SBDH data have shown comparable trends to our results. Wang et al [14] found that 49% of patients enrolled in a lung cancer cohort had smoking information captured in their EHR’s structured data. Navathe et al [13] assessed the prevalence of SBDH in EHR’s structured data and administrative claims. Smoking and alcohol abuse were reported for 15% and 8% of patients, respectively. Other domains, such as housing instability and poor social support, were reported for less than 1% of their patients. In another study, assessment of insurance claims and EHR data of older adults provided relatively similar results with only 0.03% of claims and 0.06% of EHR’s structured data providing information related to lack of social support [12,32]. Similarly, Torres et al [15] found SBDH codes being underutilized for tracking social needs using a national sample of hospital discharges (ie, <7% of discharges in any demographic or payer subgroup). Finally, Oreskovic et al [16] developed a systematic approach to identify psychosocial risk factors within any part of a patient’s EHR record and detected an average of approximately 14 SBDH-related codes/words per Medicaid enrollee.

A few studies have also assessed the value of EHR’s unstructured data to identify SBDH factors and findings vary across studies. Our findings were comparable with those of the study by Navathe et al [13] for housing issues, where 2% of their patients had information on housing instability in their EHR’s unstructured data. In contrast, our figures were much lower than their findings of 16% for social connection/isolation using unstructured EHR data [13]. Another study revealed that 29.8% of their patients had a lack of social support documented in the EHR’s unstructured data [12,32]. Similar to previous studies [13], a small group of our patients had at least one note containing mentions of select SBDH domains; however, although these numbers were low, they were much higher than SBDH factors identified using EHR’s structured data. The considerable differences of findings across studies assessing EHR’s unstructured data for SBDH might be because of various reasons, such as differences in subpopulations of interest as well as variations in text-mining methods and other NLP techniques (eg, developing different phrases and concepts referring to the same SBDH domain). Using common phrases addressing SBDH and sharing EHR free text manually tagged for specific SBDH domains can potentially help in reducing the NLP-derived variations [32].

### Harmonizing the Collection of Social and Behavioral Determinants of Health in Electronic Health Records

Major efforts are underway to increase the standardized vocabulary and content of EHR data across the nation [33,34], which would eventually impact the quality and coverage of SBDH documentation in EHRs. For example, the Centers for Medicare and Medicaid Services (CMS) required the collection of demographic information, including race, ethnicity, and preferred language, and smoking status as the core measures in stage 1 of the meaningful use (MU) program [35]. In addition, CMS now requires that all in-scope clinicians apply standardized processes and definitions within their certified EHR to screen for and document SBDH concerning food security, employment, and housing [36]. Such initiatives are fiscally backed by Medicare and might offer a successful framework for the collection of consistent SBDH data across EHRs.

Despite advancements in harmonizing and incentivizing SBDH collection within EHRs, health care organizations and clinical providers have several competing priorities, which might result in a modest rate of data being recoded for these variables [3,31]. For instance, in our study, data related to alcohol use and smoking status were mostly collected after 2013, a period that required complying with CMS-MU program. But only approximately 9% of our patients had information regarding alcohol use and around 32% had information regarding smoking status in their structured EHR. An explanation for the incomplete SBDH data could be that collecting SBDH in structured EHR fields increases the workload of clinicians who are already overwhelmed with collecting other data types used for measuring clinical performance and health outcomes.

Another factor limiting the harmonization of SBDH within EHRs is the lack of comprehensive metadata for SBDH-related surveys that are stored within the EHR’s data warehouse (eg, Epic’s flowsheet). In this study, EHR-embedded custom-made questionnaires contained valuable information on specific SBDH domains, but the identification process of individual SBDH factors in those questionnaires was cumbersome and time-consuming. Creation of institutional-wide data dictionaries to capture and share metadata of existing EHR questionnaires addressing SBDH may propel the extraction of specific SBDH-related data from such questionnaires [7]. SBDH-specific data dictionaries could also be used to categorize SBDH questionnaires by function (eg, inpatient nursing assessment and ambulatory screening) and provide an aggregate count of utilization by location, department, and provider type. In addition, our study and similar assessments present variations in the content and quality of SBDH questionnaires and documentation within EHRs [21,37], hence increasing the need for data dictionaries to reduce ambiguity in distinguishing SBDH domains of interest for research and quality improvement processes.

### Potential Use of Natural Language Processing in Extracting Social and Behavioral Determinants of Health From Electronic Health Records

Although EHR vendors have started deploying modules to collect SBDH data at the point of care, common standardized formats are not adopted to encode this information in EHRs as structured data [3,31,33]. In such circumstances, development of EHR-based NLP (ie, text mining) techniques that extract data from unstructured EHRs would result in the identification of patients at risk and assist providers in focusing their resources on assessment of the needs of vulnerable patients (eg, prescreening for SBDH surveys). The use of NLP (ie, text mining) techniques might also reduce provider workload and help with identifying patients at risk of social and behavioral risk factors. In this study, we evaluated the use of rule-based text-mining methods and explored the utility of pattern-based techniques [12,14,30] to extract selected domains from unstructured data. We investigated the coverage and accuracy of these methods among various clinical notes authored by different providers. Similar to previous studies, the majority of notes containing SBDH were authored by physicians [13]. Future studies should measure the association of notes and provider types with captured data on SBDH in EHRs’ free text, hence enhancing the text-mining process by targeting the most valuable notes.

The reported text-mining findings in our study were based on the occurrences of specific linguistic patterns (eg, phrases, such as homelessness) within clinical notes. The results showed promising accuracy and efficiency but at the expense of coverage. Linguistic patterns related to SBDH helped us develop an efficient NLP pipeline; however, advanced study (eg, manual annotation of SBDH in a large body of text) is needed to evaluate the rate of false negative cases. In addition, deterministic information found in the structured fields (including embedded questionnaires) can be used to create valuable training and validation datasets for machine learning experiments [38]. Advanced NLP techniques would help to automatically extract highly associated linguistic patterns from the notes of specific cohorts and utilize those patterns to improve SBDH coverage.

### Implications for Population Health Analytics

EHRs have been proposed as data sources of SBDH for population health purposes [39,40]. Previous studies have shown a significant role for EHR-derived data in improving population health analytics and risk stratification efforts [41-46]. A growing number of studies have also shown the added value of EHR-derived SBDH data in supporting population health management efforts, such as care coordination [47,48]. However, certain challenges should be addressed to make EHRs a reliable source of SBDH data on a population-level: immaturity of EHRs to collect and organize SBDH data [31,32,49], EHR data quality issues including missing data [50,51], and the need for complex methods to extract SBDH from EHR’s free text [12,30-32]. Extracting SBDH data from non-EHR data sources (eg, health information exchanges and geographical information systems) should be further assessed as an approach to compensate for missing SBDH data in EHRs [52]. Finally, as population and public health informatics are merging efforts toward a common goal of improving health outcomes for all [53-55], identifying SBDH factors of high-risk patients using EHRs will be a key in addressing community-level health disparities [19,20].

### Limitations

Our study has several limitations: (1) our results were driven by the underlying EHR data of a specific multilevel academic health care system. Other health care organizations may find data on SBDH captured and collected at different rates depending on the characteristics of their patient population, workflow, EHR use, and other system or policy factors, (2) our study used ICD codes to identify information stored as structured data; however, other coding terminologies (eg, LOINC, SNOMED) have also addressed those determinants of health. Investigation of information captured in EHRs using different coding systems might help identify more information stored as structured data, (3) our study focused on data captured before 2018; however, because of the trends in value-based payment models and policy requirements, a rise in collection of SBDH information within EHR settings is likely to have already begun, and (4) our NLP approach (ie, text-mining techniques) used a pattern matching algorithm with no measure of false negative rates, which might have limited our ability to detect higher number of patients with mentions of SBDH; thus, future studies should focus on developing robust NLP methods with high measures of recall (sensitivity) and precision (specificity) to extract all types of phrases used to describe SBDH from EHR’s unstructured data.

### Conclusions

To our knowledge, this study is the first attempt by a major health care system to provide an investigator-friendly report of SBDH data from its EHR. We assessed rates of SBDH collection within structured EHR data of approximately 5.4 million patients and the unstructured EHR data of approximately 1.2 million patients to reduce possible sampling errors. Data were also collected from a variety of health care settings, which helped avoid the possibility that physicians in one setting might have habitually failed to collect SBDH data. Findings of this study can also serve as a baseline for future studies using advanced NLP approaches [56] to extract more complex SBDH domains from EHRs. We hope that our results will inform providers, researchers, and health care systems to understand the value of EHRs in capturing SBDH data, provide support to informaticians to advance the standardization of EHR-based tools and terminologies for SBDH data collection, and help decision makers to plan for the integration of SBDH in population health management efforts.

## Appendix

Multimedia Appendix 1 Example of available codes and phrases for different subdomains of housing issues.

Multimedia Appendix 2 Example of phrases developed for various aspects of social connection/isolation.

Multimedia Appendix 3 Other data collection methods for selected SBDH in an EHR's structured data.

Multimedia Appendix 4 Characteristics of EHR questionnaires capturing data on selected SBDH.

Multimedia Appendix 5 An overview of EHR data availability timeline across different facilities of the health care system.

Multimedia Appendix 6 Comprehensive list of SBDH domains.

## Abbreviations

CMS: Centers for Medicare and Medicaid Services
EHR: electronic health record
ICD-10: International Classification of Diseases–10th Revision
ICTR: Institute for Clinical and Translational Research
LOINC: logical observation identifiers names and codes
MU: meaningful use
NAM: National Academy of Medicine
NCATS: National Center for Advancing Translational Sciences
NIH: National Institutes of Health
NLP: natural language processing
SBDH: social and behavioral determinant of health
SNOMED: systematized nomenclature of medicine
SQL: structured query language
