# Supplementary material for: Assessing the Availability of Data on Social and Behavioral Determinants in Structured and Unstructured Electronic Health Records: A Retrospective Analysis of a Multilevel Health Care System
Source: JMIR Med Inform. 2019 Aug 2;7(3):e13802. doi: 10.2196/13802 (PMC6696855; doi:10.2196/13802)
Supplement: Multimedia Appendix 1 [file medinform_v7i3e13802_app1.docx]

**Appendix Table 1. Example of Available Codes and Phrases for Different Sub-Domains of Housing Issues**

| **ICD10** | **SNOMED** | **LOINC** | **Public Health Surveys  and Instruments** | **Literature Review Phrases** | **Manual Tagging** |
| --- | --- | --- | --- | --- | --- |
| Homelessness (Z59.0) | Length of time homeless (442244004) | Housing status (71802-3) | Screening instruments (Housing Vital Sign AND PRAPARE^a^); What is your housing situation today? | Homeless | Living in facility |
| Problems related to housing & economic circumstances, unspecified (Z59.9) | Homeless (32911000) |  | PRAPARE: What is your housing situation today? I chose not to answer this question | Homelessness | Assistance with housing |
| Other problems related to housing & economic circumstances (Z59.8) | Homeless single person (160700001) |  | Public (section 8) housing/temporary housing/housing subsidies | No home | House will be renovated |
| Discord with neighbors, lodgers and landlord (Z59.2) | Homeless Family (105526001) |  | PRAPARE: What is your housing situation today? I have housing | Houseless |  |
| Inadequate housing (Z59.1) | Housing lack (266935003) |  | Screening instrument (Housing Vital Sign): I have housing today, but I am worried about losing housing in the future | Unhoused |  |

^a^ PRAPARE: Protocol for Responding to and Assessing Patients’ Assets, Risks, and Experiences
