# Supplementary material for: Assessing the Availability of Data on Social and Behavioral Determinants in Structured and Unstructured Electronic Health Records: A Retrospective Analysis of a Multilevel Health Care System
Source: JMIR Med Inform. 2019 Aug 2;7(3):e13802. doi: 10.2196/13802 (PMC6696855; doi:10.2196/13802)
Supplement: Multimedia Appendix 2 [file medinform_v7i3e13802_app2.docx]

**Appendix Table 2. Example of Phrases Developed for Various Aspects of Social Connection / Isolation**

| **Lack of Social Support (Social Isolation) or  At Risk for Social Isolation (N=258)** | | | **In Need of Social Support Services (N=166)** | | |
| --- | --- | --- | --- | --- | --- |
| **Green Phrases**^a^ **(N=119)** | **Yellow Phrases**^a^ **(N=113)** | **Red Phrases**^a^ **(N=26)** | **Green Phrases**^a^ **(N=106)** | **Yellow Phrases**^a^ **(N=44)** | **Red Phrases**^a^ **(N=16)** |
| No household member renders care | Alone | Power of attorney | Patient has no care provider | Patient should not live alone | Persons encountering health services in other circumstances |
| Lonely | lives alone | High expressed emotional level within family | No help | It is not safe for patient to live alone | Community living |
| No social support | Lives by herself | Usually attends church, temple | Patient has functional limitations | Lack of assistance | Live in a shelter |
| No family supports | Lives by himself | Social interaction almost daily | Unable to walk | Lack of transportation | Live in a community housing |

^a^ Color-coding represents relevance of developed phrases to the SBDH domain of interest
(green, yellow, and red: ranging from most relevant to the least relevant, respectively)

Color coding presented the relevance of phrases to the SBDH domains. Our text mining algorithms were mostly built on green phrases. We did not assess the rate of false positives for different categories of phrase.
