# Supplementary material for: Assessing the Availability of Data on Social and Behavioral Determinants in Structured and Unstructured Electronic Health Records: A Retrospective Analysis of a Multilevel Health Care System
Source: JMIR Med Inform. 2019 Aug 2;7(3):e13802. doi: 10.2196/13802 (PMC6696855; doi:10.2196/13802)
Supplement: Multimedia Appendix 3 [file medinform_v7i3e13802_app3.docx]

**Appendix Table 3. Other Data Collection Methods for Selected SBDH in EHR’s Structured Data**

| **Collection Method** | **Completeness Rate** | **Facility/Provider** | **Dates** |
| --- | --- | --- | --- |
| **Patient Address** | | | |
| Hospital Account | 73% of the 7.3 million hospital accounts | Inpatient facilities, primarily in imaging and physical therapy services at two community hospitals | 2013 – Current |
| Temporary Address | 0.3% out of 5.4 million patients | All facilities at the time of registration | 2003 – Current |
| Billing Address | 99% of 2.1 million patient accounts where the guarantor is the patient | All facilities at the time of registration | 2003 – Current |
| Claims Processing Address | 100% of 4.9 million claims where the patient was listed |  | 2013 – Current |
| Insurance Coverage Address | 92% of 3.1 million coverage records where the subscriber is also the patient | All facilities at the time of registration | 2006 – Current |
| Home Health Encounters and Episodes | 99% of the 41,604 home care encounters | At the time of the Home Health scheduling encounter | Sep 2015 – Current |
| MyChart Patient Proxy Addresses | 79% of the 30,410 MyChart proxy records | MyChart proxies are MyChart users who log in to MyChart on behalf of a patient. In this case, proxies are not patients. Note that address is not complete for all records. |  |
| Communications for Specific Encounters | 100% of the 747,616 encounter-based communications | Clinicians have the ability to track communications associated with specific encounters. The most common provider types are physicians and medical assistants. | 2013 – Current |
| Social Work Assessment Questionnaire | 7% of the 14,129 times that the questionnaire was completed, patient and/ or primary care giver address was provided | This questionnaire focuses on the patient’s social, economic, safety, and psychological well-being. Captured at inpatient emergency services and admissions by social workers at two Johns Hopkins affiliated hospitals. | Jul 2014 – Current |
| Maltreatment Assessment: Father’s address / Mother’s address | 7% of the 14,129 times the template was used and the question was answered | JHH Pediatric ED, other pediatric clinics, by social workers | Apr 2013 – Current |
| **Ethnicity** | | | |
| Transplant Organ Donors | 50% of the 4,647 human transplant donors in documented in the EHR |  | Aug 1996 – Current |
| Ethnicity Questionnaire | In 20% of the 2,471 times that the questionnaire was completed, the question “What is your race/ethnicity” was answered. The choices for selection were Black, Hispanic, and Neither Black Nor Hispanic. | Inpatient encounters at Johns Hopkins Hospital by registered nurses and case workers. | Jan 2016 – Oct 2016 |
| Ethnicity Origin Questionnaire | In 5% of the 3,558 times that the questionnaire was completed, the questions “Father’s Ethnic Origin” and “Mother’s Ethnic Origin” were answered. The choices for selection were:  Caucasian, Hispanic, Black, Asian, Middle Eastern, and Other. | Two ophthalmology clinics by technicians | Apr 2013 – Sep 2017 |
| Pregnancy / Delivery Episodes: Father’s Ethnicity | 0.1% of the 109,574 combined pregnancy episodes  The choices for selection are:  Not Hispanic or Latino, Hispanic or Latino, Unknown, Patient Refused. | Two Johns Hopkins Community Physicians OBGYN outpatient clinics | 2015 – Current |
| **Race** | | | |
| Home Health | 84% of the 40,301 Home Health episodes documented in the EHR. | For Home Health encounters only, patients can select multiple races to self-identify. The selections differ from the most common collection method:  American Indian or Alaska, Native, Asian, Black or African-American, Hispanic or Latino, Native Hawaiian or Pacific Islander, and White. | 2004 – Current |
| BCRA (Bay Cities Racing Association) Race | 0.05% of the 113 million BCRA encounters | Race is also collected at outpatient and inpatient encounters at breast imaging, ultrasound, and MRI clinics throughout the institution. One race per patient is selected, and race selection is restricted to the following: White, African American, Hispanic, Chinese American, Japanese American, Filipino American, Hawaiian Pacific Islander, and Other Asian American. | May 2013 – Current |
| Transplant Organ Donors | 44% of the 4,602 organ donors | Multiple races are captured for each donor from the same selections as available during the patient registration process | 1996 – Current |
| Race Questionnaire | 7.4% of the 1,748 times the questionnaire was used at an HIV clinic encounter | Race is documented for patients seen in the Johns Hopkins Hospital HIV Clinics, specifically collected by social workers and case managers. Only one race can be selected. Selections are restricted to the following: White, Black or African American, Asian  Native Hawaiian or Other Pacific Islander, American Indian or Alaska Native, and Other (Comment) | Apr 2013 – Current |
