# Supplementary material for: Assessing the Availability of Data on Social and Behavioral Determinants in Structured and Unstructured Electronic Health Records: A Retrospective Analysis of a Multilevel Health Care System
Source: JMIR Med Inform. 2019 Aug 2;7(3):e13802. doi: 10.2196/13802 (PMC6696855; doi:10.2196/13802)
Supplement: Multimedia Appendix 4 [file medinform_v7i3e13802_app4.docx]

**Appendix Table 4. Characteristics of EHR Questionnaires Capturing Data on Selected SBDH**

| **Questionnaire Template** | **Content Area** | **Completeness Rate** | **Facility** | **Provider** | **Collection Date** |
| --- | --- | --- | --- | --- | --- |
| **Social Support** | | | | | |
| Nursing Assessment – Psychosocial | Psychosocial | 1,026,988 completed questionnaires; 944,829 (92%) answered the question. | 1. Inpatient Units at a County General Hospital and 2. Suburban a Community Hospital | Registered Nurses, Licensed Practical Nurses, Case Managers | May 2013 – Current |
| Emergency Department Assess Head to Toe | Psychosocial | Of the 237,143 completed questionnaires; 92,486 (39%) answered the psychosocial question | Inpatient Social History Clinical Decision Unit at a Johns Hopkins Community Hospital | Registered Nurses | Oct 2013 – Current |
| Emergency Department Nursing Assessment | Psychosocial | 217,954 completed questionnaires; 204,877 (94%) answered the question. | Emergency Medicine Inpatient units at Main Johns Hopkins Hospital and a Johns Hopkins Affiliated Hospital | Registered Nurses | Dec 2015 – Current |
| Emergency Department Nursing Assessment | Psychosocial | 278,084 completed questionnaires; 169,631 (61%) answered the question. | Emergency Units at a County General Hospital, and Two Johns Hopkins Community Hospitals | Registered Nurses | Dec 2015 – Current |
| Emergency Department Pediatrics Assessment | Psychosocial | 131,134 completed questionnaires; 93,105 (71%) answered the question. | Pediatrics Emergency Units at Johns Hopkins Main Hospital, a County General Hospital, and Two Johns Hopkins Community Hospitals | Registered Nurses | Dec 2015 – Current |
| OR and PACU Flowsheet | Psychosocial | 147,694 completed questionnaires; 82,709 (56%) answered the question. | Inpatient Post-Anesthesia Units at Johns Hopkins Main Hospital | Registered Nurses | Sep 2015 – Current |
| Inpatient, Occupational Therapy New Home Setup | Social Support Available at Discharge | 131,948 completed questionnaires; 47,501 (36%) answered the question. | Inpatient Orthopedic and Surgical Floors at a Johns Hopkins Affiliated Hospital and a Community Hospital | Occupational Therapists | Jun 2015 – Current |
| Inpatient, Obstetrics Postpartum Assessment | Recent Loss, or Change in Status? (Includes loss of social status, job, divorce, death, demotion, etc.) | 135,587 completed questionnaires; 120,672 (89%) answered the question. | Inpatient Units at a Johns Hopkins Community Hospital and a County Hospital | Registered Nurses | May 2013 – Current |
| Inpatient Spiritual Care Interventions | Spiritual/Social Network | 116,719 completed questionnaires; 68,864 (59%) answered the question. | Inpatient Units at a Johns Hopkins Community Hospital and a County General Hospital | Chaplains | May 2013 |
| Pediatrics Screening | Personal-Social Relationship with Peers, Parents, Siblings or Socially Withdrawn and Decreased Interaction | 144,659 completed questionnaires; 85,349 (59%) answered the question. | Johns Hopkins Community Physicians Pediatric Clinics | Medical Assistants, Physicians, Nurse Practitioners | Apr 2013 – Current |
| Social History; Screening, Brief Intervention and Referral to Treatment | Marital Status / Need to Improve Relationships with Family/ Social Network and Participation in Social Activities | 2,015 completed questionnaires; 1995 (99%) answered the question. | Inpatient Units at a Johns Hopkins Affiliated Hospital | Peer Recovery Coaches | Jul 2017 – Current |
| Emergency Department Social Work Suicide/ Homicide Assessment | Relationship and Social Support Status | 15,101 completed questionnaires; 14,648 (97%) answered the question. | Inpatient Units at two Johns Hopkins Community Hospitals | Social Workers, Therapists | Jun 2013 – Current |
| Emergency Department Social Work Assessment | Support System’s Name and Information | 14,481 completed questionnaires; 12,743 (88%) answered the question. | Emergency Departments at a Johns Hopkins Community Hospital and an Affiliated Hospital | Social Workers, Therapists | Jul 2014 – Current |
| **Housing Issues** | | | | | |
| Housing/Utility Voucher | Housing Assistance Screening and Referral | 217 completed questionnaires; 97 (44%) housing financial assistance was awarded. | HIV Clinics | Social Workers, Case Managers | Mar 2017 – Current |
| Abuse/Neglect Screen | Homelessness Assessment (as a part of a larger evaluation) | 12,058 completed questionnaires; 11,575 (96%) homelessness was answered (yes or no). | 1. The Infusion Center at a Johns Hopkins Community Hospital  2. The Breast Clinic at a Johns Hopkins Community Hospital  3. The Physical Therapy Department at a Johns Hopkins Community Hospital  4. A County General Wound Center. | Registered Nurses, Physical Therapists, Wound Ostomy, Continence Nurses | Jun 2013 – Current |
| Social History Questionnaire Screening, Brief Intervention, and Referral to Treatment | Screening for Assistance with Finding Housing (as a part of a larger evaluation) | 1,900 completed questionnaires; 1824 (96%) answered the question (yes or no). | 1. Emergency Services and 2. BMC Chemical Dependency Units at a Johns Hopkins Affiliated Hospital | Peer Recovery Coaches | Jul 2017 – Current |
| ED Triage Abuse Indicators and Resource Planning | Patient Indicators, Resource Planning, and Outcomes for Shelter, Transportation, and Clothing | 713,702 completed questionnaires; 39,254 (5.5%) answered housing questions. | 1. Inpatient and  2. Emergency Units | Registered Nurses | May 2013 – Current |
| Chemical Dependence Unit Admission Screen | Homelessness | 15,056 completed questionnaires; 2,258 (15%) homelessness was answered (yes or no). | Clinical Decision Unit at a Johns Hopkins Community Hospital | Registered Nurses | Jul 2014 – Current |
| Ambulatory Priority Access Primary Care Screen | Housing Situation | 1,116 completed questionnaires; 78 (7%) answered the housing question. | A Johns Hopkins Community Physicians Internal Medicine Clinic | Physicians, Medical Assistants | Apr 2015 – May 2017 |
| Adult Admission General Intake Form | Homelessness | 77,230 completed questionnaires; 27030 (35%) homelessness was answered (yes or no). | 1. Inpatient Units at a Johns Hopkins Community Hospital  2. Inpatient Units at a County General Hospital | Registered Nurses | May 2013 – April 2016 |
| Pediatric/Newborn General Intake Form | Homelessness | 1,067 completed questionnaires; 587 (55%) homelessness was answered (yes or no). | 1. Johns Hopkins Main Hospital  2. Johns Hopkins Affiliated Hospital | Social Workers | 2016 – Current |
| Psychiatry Social Work Assessment | Living Arrangement | 4,913 completed questionnaires; 4,422 (90%) answered the question. | Inpatient Psychiatry at a Johns Hopkins Affiliated Hospital | Social Workers | Sep 2015 – Current |

OR: Operation Room; PACU: Post-Anesthesia Care Unit
