# Supplementary figures and images for: Assessing the Availability of Data on Social and Behavioral Determinants in Structured and Unstructured Electronic Health Records: A Retrospective Analysis of a Multilevel Health Care System
Source: JMIR Med Inform. 2019 Aug 2;7(3):e13802. doi: 10.2196/13802 (PMC6696855; doi:10.2196/13802)

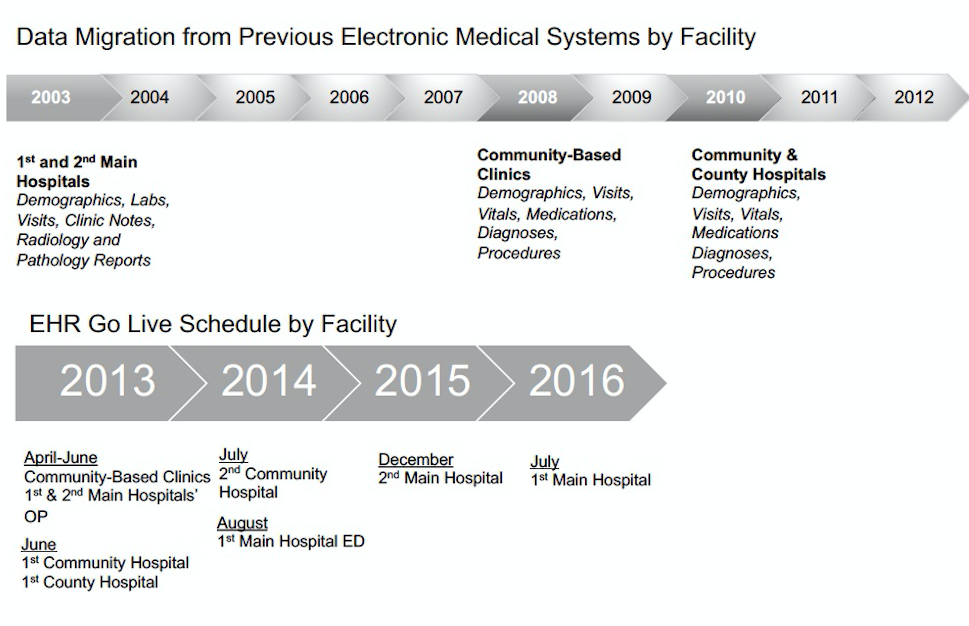

Supplement: Multimedia Appendix 5 [file medinform_v7i3e13802_app5.png]

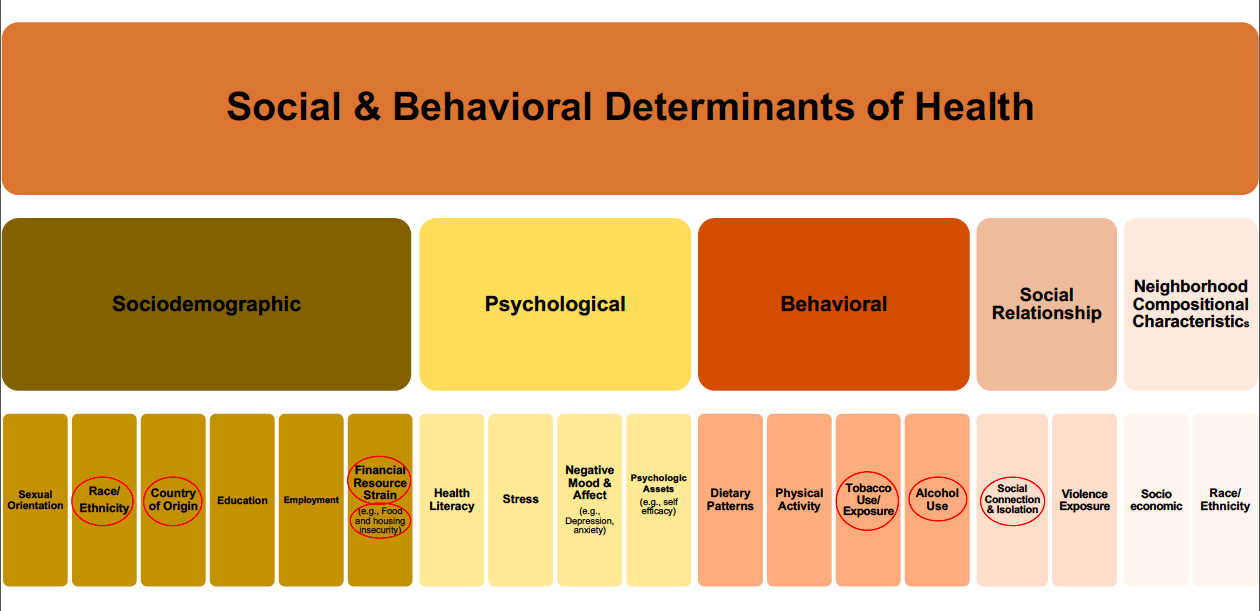

Supplement: Multimedia Appendix 6 [file medinform_v7i3e13802_app6.png]
